# Effects of Disturbance Intensity and Frequency on Bacterial Community Composition and Function

**DOI:** 10.1371/journal.pone.0036959

**Published:** 2012-05-14

**Authors:** Mercè Berga, Anna J. Székely, Silke Langenheder

**Affiliations:** Department of Ecology and Genetics/Limnology, Uppsala University, Uppsala, Sweden; Utrecht University, The Netherlands

## Abstract

Disturbances influence community structure and ecosystem functioning. Bacteria are key players in ecosystems and it is therefore crucial to understand the effect of disturbances on bacterial communities and how they respond to them, both compositionally and functionally. The main aim of this study was to test the effect of differences in disturbance strength on bacterial communities. For this, we implemented two independent short-term experiments with dialysis bags containing natural bacterial communities, which were transplanted between ambient and ‘disturbed’ incubation tanks, manipulating either the intensity or the frequency of a salinity disturbance. We followed changes in community composition by terminal restriction fragment analysis (T-RFLP) and measured various community functions (bacterial production, carbon substrate utilization profiles and rates) directly after and after a short period of recovery under ambient conditions. Increases in disturbance strength resulted in gradually stronger changes in bacterial community composition and functions. In the disturbance intensity experiment, the sensitivity to the disturbance and the ability of recovery differed between different functions. In the disturbance frequency experiment, effects on the different functions were more consistent and recovery was not observed. Moreover, in case of the intensity experiment, there was also a time lag in the responses of community composition and functions, with functional responses being faster than compositional ones. To summarize, our study shows that disturbance strength has the potential to change the functional performance and composition of bacterial communities. It further highlights that the overall effects, rates of recovery and the degree of congruence in the response patterns of community composition and functioning along disturbance gradients depend on the type of function and the character of the disturbance.

## Introduction

Disturbances are discrete events in time that cause changes in resources or in the physical environment and, thus, influence community structure. They are known to lead to changes in species richness [1], [2] and to impair ecosystem functioning [3], [4]. Bacteria are the main contributors to the flow of carbon and nutrients in nature [5] and therefore important key players in ecosystems. Thus, more knowledge is needed towards the understanding of the effect of disturbances on bacterial communities.

In general, a community can respond to disturbances in different ways regarding composition and function [6], [7]. With the main focus on microbial community composition, a community can be resistant, i.e., withstand disturbances without any changes in composition [8]. It can be resilient, i.e.; recover from a disturbance-induced change and regain the original state [9]. More types of responses can be observed when also functions are included in the studies [10], [11]. A community is functionally redundant if a change in community composition is not accompanied by a change in a function provided by the communities after the disturbance [12], [13]. Alternatively, a community might have a high functional plasticity: only functions but not community composition change after the disturbance [14], with the possibilities of functional recovery or functions remaining altered once the disturbance ceases. Thus, there is no general pattern for the community composition – function relationship. Moreover, some studies have suggested that this relationship might depend on the level of the studied function [15], [16].

Allison and Martiny (2008) reviewed the available literature on soil microbial communities and found that microbial composition is generally sensitive to disturbances, and that most microbial communities do not return rapidly to their original state, i.e. show low degrees of resilience. However, not many studies have addressed resilience [6]. It has been suggested that the effect of disturbance on bacterial community function might depend on the function measured, the type of disturbance [17], as well as the temporal patterns of the disturbance [18].

Most of the described patterns regarding responses to disturbances are based on macroorganisms and there is currently a lack of understanding about how bacterial communities respond including their ability to resist and recover from disturbances that differ in strength. It is therefore essential to investigate effects of differences in disturbance intensity and frequency on bacterial communities using controlled experimental studies. Salinity, being a physiological stress factor, has been shown to be a strong structuring factor for bacterial communities, affecting functional performance and growth rates and leading to shifts in composition [19]–[22]. As such, it has been shown that salinity is the most important factor explaining global distribution patterns of bacteria as well as other microorganisms [23], [24]. Here, we implemented two independent short-term experiments where we manipulated either the intensity or the frequency of a salinity disturbance affecting a rock pool bacterial community. Moreover, bacterial communities in rock pools experience natural fluctuations in salinity at more or less regular intervals so that communities inhabiting them are frequently exposed to salinity disturbances and may therefore have some degree of tolerance to salt [22], [25]. The experiments were implemented using dialysis bags that were transplanted either into tanks with several levels of increased salinity (intensity experiment) or swapped between one high salinity tank and the control tank at different intervals (frequency experiment). In both cases bags were moved back to the control tanks after the disturbance period to measure the ability of short-term recovery after the applied disturbance regime. To be able to detect rapid changes in response to the disturbance, we specifically analyzed the composition of the active fraction of bacterial community by 16S rRNA based terminal restriction fragment analysis [26]. At the same time we studied several community functions.

Here we specifically address: (1) How bacterial community composition (BCC) and several functions provided by the communities change along a gradient of increasing disturbance intensity and frequency, (2) whether resilience, i.e. the ability to recover from the disturbance is related to disturbance intensity and frequency, respectively; and finally, (3) if there are concurrent response patterns occurring in BCC and community function along the disturbance strength gradient. Since salinity fluctuations occur naturally in rock pools, we hypothesize that bacterial community composition as well as function will be resistant to low and infrequent salinity increases. Moreover, if resistance is not found, we hypothesize that communities will recover to a greater extent at low compared to intermediate or high levels of disturbance intensity and frequency. Finally, we hypothesize that functional redundancy among taxa in the community will be high so that disturbances will have stronger effects on community composition than on function.

## Materials and Methods

### Ethics Statement

No specific permits were required for the sampling and activities performed in the study.

### Experimental set-up

Water from a freshwater rock pool (temperature: 4.7°C, salinity: 0.2 psu) was collected in October 2009 on the island of Gräsö, Sweden (60.498 N, 18.430 E). Water (250 L) was taken and stored in a constant temperature room (4°C) until the experiment was started one month later. The water was either filtered through A/E glass fiber filters (1.2 μm, Pall Life Science, NY, USA) to remove big particles and used to fill 6 tanks of 40 L, or through GF/F filters (0.7 μm, Whatman, UK) to additionally remove bacterial grazers and used to fill dialysis bags. Dialysis membrane tubing with a length of 50 cm and a width of 45 mm (ZelluTrans, Dialysis tubular membrane T4, Molecular Weight Cut Off: 12000–14000 Daltons, Carl Roth, Karlsruhe, Germany) were filled with 150 mL water. The tubing was closed at both ends with universal dialysis tubing closures (Spectrum Laboratories, Inc., The Netherlands). Two transplant experiments were implemented where either disturbance intensity (Experiment 1) or disturbance frequency (Experiment 2) were manipulated. Both experiments were implemented at constant temperature (20°C) and incubated in the dark. Salinity equalization and sterility were monitored during the experiment.

#### Experiment 1: Disturbance intensity

Artificial brackish water solution [27] was added to six tanks to create a gradient of salinity from original salinity (0.2 psu) to 20 psu as follows: 0.2 psu (control), 3 psu, 5 psu, 10 psu, 15 psu and 20 psu. After an initial period of acclimatization in the control tank for 8 hours, triplicate bags were moved to the respective treatments and incubated for 36 hours, and subsequently moved back to the control tanks where they were incubated for another 36 hours before the experiment was terminated. Samples were taken at the end of the incubation in the treatment tanks to account for effects at the end of the disturbance period, and at the end of the experiment to account for potential effects of short-term recovery. The two sampling times are called “after disturbance” and “at the end”, respectively.

#### Experiment 2: Disturbance frequency

For this experiment, dialysis bags were swapped at different frequencies between the control and 20 psu tank during a total period of 96 hours. As in the disturbance intensity experiment, the bags were first acclimatized for 8 hours in the control tank. During the following 64 hours the bags were moved and incubated in the 20 psu tank for 8 hours either once, twice, three-times or four-times. Following, the bags were sampled to investigate the immediate disturbance effects (after disturbance) and then incubated for another 24 hours in the control tank before the final sampling was performed (at the end). It is important to note that the differences in the applied disturbance frequencies resulted in changes in the total duration of the applied disturbance as well as the length of the recovery period.

Samples were taken by emptying approximately one third of the content of each bag (40–50 mL) into a sterile container, keeping the remaining volume of the bag untouched by adding an extra closure. For each sample, subsampling was done for measurements of the active bacterial community composition and for different functions: (1) total bacterial abundance; (2) overall bacterial production measured as leucine incorporation rates; (3) the metabolic capacity of the community to utilize a variety of carbon substrates (total Carbon Substrate Utilization Rates (tCSURs) estimated from Biolog © Ecoplates); (4) functional diversity, i.e., the number of carbon substrates utilized; and finally, (5) changes in substrate guild preferences.

**Figure 1:**
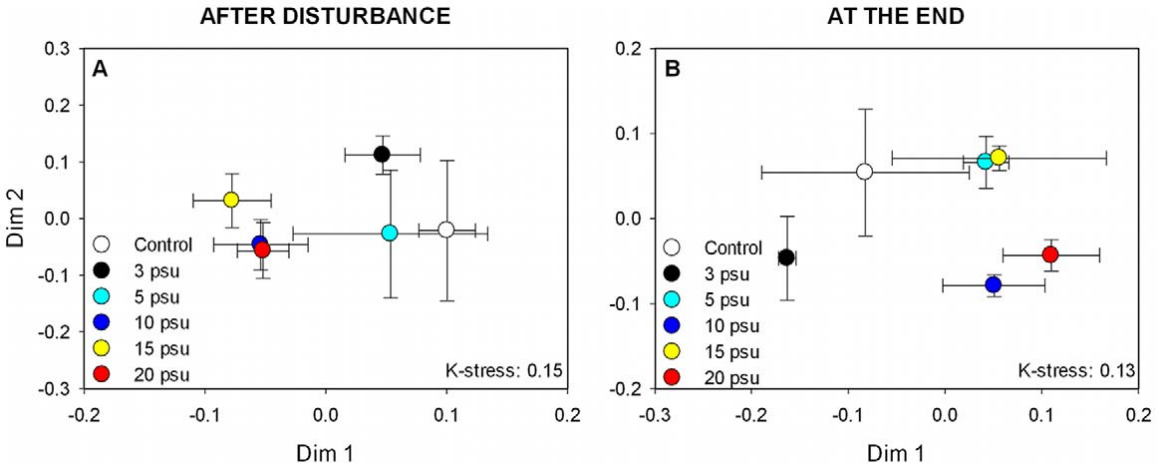
NMDS plots showing the changes in BCC in relation to disturbance intensity (Experiment 1). NMDS are based on Bray-Curtis distances according to bacterial community composition patterns (t-RFLP), after the disturbance (A) and at the end of the experiment (B). Error bars indicate standard errors.

**Figure 2:**
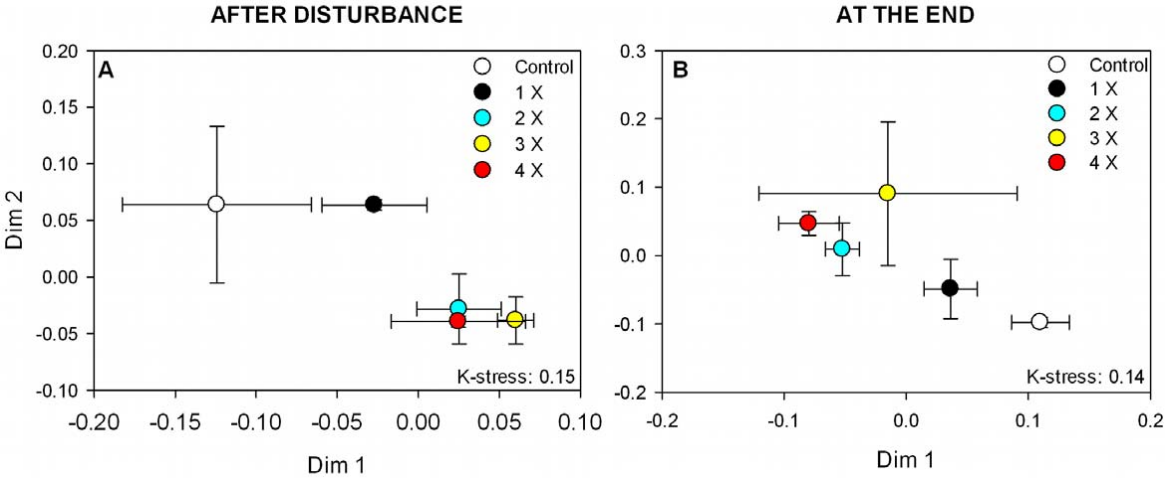
NMDS plots showing the changes in BCC in relation to disturbance frequency (Experiment 2). NMDS are based on Bray-Curtis distances according to bacterial community composition patterns (t-RFLP), after the disturbance (A) and at the end of the experiment (B). Error bars indicate standard errors.

### Sample analyses

#### Bacterial community composition

Bacterial DNA and RNA were co-extracted from filters of 30 mL applying the protocol #3 of the Easy-DNA™ kit (Invitrogen, Carlsbad, CA, USA) with an extra bead-beating step using 0.2 g of 0.1 mm silica beads. For the reverse transcription, DNA was removed from the DNA–RNA extracts by treatment with DNase I (Invitrogen) for 15 minutes at room temperature and the reverse transcription of the RNA fraction was conducted using the RevertAid^TM^ H Minus First Strand cDNA Synthesis Kit (Thermo Fisher Scientific, Waltham, MA, USA) with random hexamer primers in accordance to the manufacturers instructions. Transcribed cDNA of 16S rRNA was used for further downstream PCR amplifications and terminal-restriction fragment length polymorphism (T-RFLP) analysis [28]. PCR amplification was conducted using the HEX-labeled forward bacteria-specific primer 8F (5-AGRGTTTGATCMTGGCTCAG-3) and the reverse universal primer 519R (5-GWATTACCGCGGCKGCTG-3). Each 20-μL reaction consisted of 1 U Biotaq^TM^ DNA polymerase (Bioline, London, UK), 200 nM of each primer, 250 µM of each dNTP (Invitrogen) 1.5 mM MgCl (Bioline) and up to 10 ng of target DNA in 1×NH_4_ buffer (Bioline). Thermocycling was carried out with an initial denaturation step at 94°C (3 min), followed by 25 cycles of denaturation at 94°C (30 sec), annealing at 52°C (30 sec) and primer extension at 72°C (45 sec), and finalized with a 7-min extension step at 72°C. Two replicates per sample were pooled after PCR amplification and digested with Mung bean nuclease (New England Biolabs) and purified using AcroPrepTM 96-well filter plates 30 K Omega (Pall, NY, USA). Fluorescently labeled PCR products were then subjected to digestion with the restriction enzyme *Hae*III (New England Biolabs) for 16 h at 37°C. Each digestion reaction contained approximately 30 ng of DNA, 4 U of each respective restriction enzyme in a final reaction volume of 10 µL. Fluorescently labeled terminal-restriction fragments were separated and detected by an ABI 3730 capillary sequencer running in GeneScan mode (Applied Biosystems, Foster City, CA, USA) together with an internal size standard (GeneScan-500 ROX; Applied Biosystems). T-RFLP peaks were finally determined by using the program GeneMarker v 1.70 (SoftGenetics, State College, PA, USA). GeneMarker analysis included fragments with lengths ranging from 50–500 bp and an initial peak detection threshold of 100 relative fluorescent units. Peaks less than 0.5 bases apart from each other were merged. Moreover, the relative data was transformed to presence-absence data in order to estimate the number of terminal restriction fragments (T-RFs) in each sample.

#### Abundance and Functions

Bacteria were preserved by adding 37% formaldehyde to a final concentration of 4%. Bacterial counting was performed by means of a Cyflow flow cytometer (Partec, Münster, Germany), after staining the cells with 1.25 μM Syto13 solution (Invitrogen) [29]. The detector settings were optimized for fluorescence and forward scatter. Bacterial production (Leucine incorporation into protein) was obtained according to [30]. L-[4, 5-^3^H] Leucine TRK510, 139 Ci mmol^−1^ (Amersham, Buckinghamshire, UK) diluted to 15% with unlabeled L-Leucine (Sigma, St Louis, MO, USA), was added to 1.7 mL of water sample at a final L-Leucine concentration of 100 uM and incubated for 1 h. Incubations were done in duplicates and also included a control sample consisting of a sample that was inactivated by adding 100% TCA prior to the leucine addition.

To obtain an overall information of the tCSURs, the number of carbon substrates utilized and single substrate utilization rates (sCSURs), Biolog EcoPlates^TM^ (Biolog Inc. Hayward, CA, USA) [31] were used. One set, consisting of 31 carbon substrates, was inoculated per sample by pipetting 125 µL of water into the wells. Color development was measured with a microplate-reader (Ultra384, Tecan, Männedorf, Switzerland) at 590 nm, 48 h, 72 h and 120 h after inoculation. tCSUR refers to the slope of change of the average well color development (AWCD) over 72 h for each sample. AWCD is obtained by calculating the average of the well color development of all the substrates [32]. Moreover we were also interested in whether the capacities differed depending on the substrates guilds, i.e., carbohydrates, amino acids, carboxylic and acetic acids, polymers or amines and amides [33]. Thus, sCSURs were estimated by calculating the slope of change of the well color for each individual substrate. Substrates that exhibited significant differences in utilization rate between treatments were grouped into the different guilds according to their chemical properties. Finally, to obtain information of the number of carbon substrates utilized, we considered as positive responses wells with absorbance levels higher than 0.25 at the time point when the AWCD of the plate was the closest to 0.5 [34].

### Statistical analyses

For BCC analysis, the height of the 16S rRNA terminal restriction fragments (TRFs) was normalized to account for differences in total sample intensity and relative heights were calculated after applying a 1% threshold [35]. Dissimilarity matrices using Bray Curtis were calculated with the relative peak heights data and used for Non-metric Multidimensional Scaling (NMDS; XLstat, Addinsoft, Paris, France) and Analysis of Similarity (ANOSIM) using the PAST software package [36]. Finally, the number of TRFs was compared among treatments using one-way ANOVAs and subsequent post-hoc tests using PASW statistics (IBM, New York, NY, USA).

To assess changes in bacterial abundance and functions in dependence of disturbance intensity or disturbance frequency, one–way ANOVAs were performed. Bacterial production data was log transformed (log (x+1)) and tCSURs and sCSURs were square root transformed to accomplish ANOVA assumptions. Tukey's *post hoc* test was used to identify homogenous groups. To evaluate differences in sCSURs, multivariate analysis of variance (MANOVA) was implemented using the slopes of the 31 substrates as dependent variable and the respective treatment as a factor. Substrates with significant results were use for the next step and are referred to as ‘significant substrates’ throughout the manuscript. Average slopes of all the significant substrates within one guild were calculated and compared among treatments using one-way ANOVA and subsequent *post hoc* tests. Finally, the number of positive responses was compared among treatments using one-way ANOVAs and subsequent *post hoc* tests.

Mantel tests (10,000 permutations, Pearson correlations) were performed in XLstat in order to test correlations between BCC and functions at different levels: (1) correlations between BCC (Bray-Curtis dissimilarities) and the overall bacterial production (Euclidean distances of log-transformed bacterial production rate), (2) correlation between BCC and the overall metabolic capacity in terms of carbon substrate utilization (Euclidean distances of square root transformed tCSURs), and (3) correlation between BCC and the sCSURs of the significant substrates (Euclidean distances of sCSURs).

**Figure 3:**
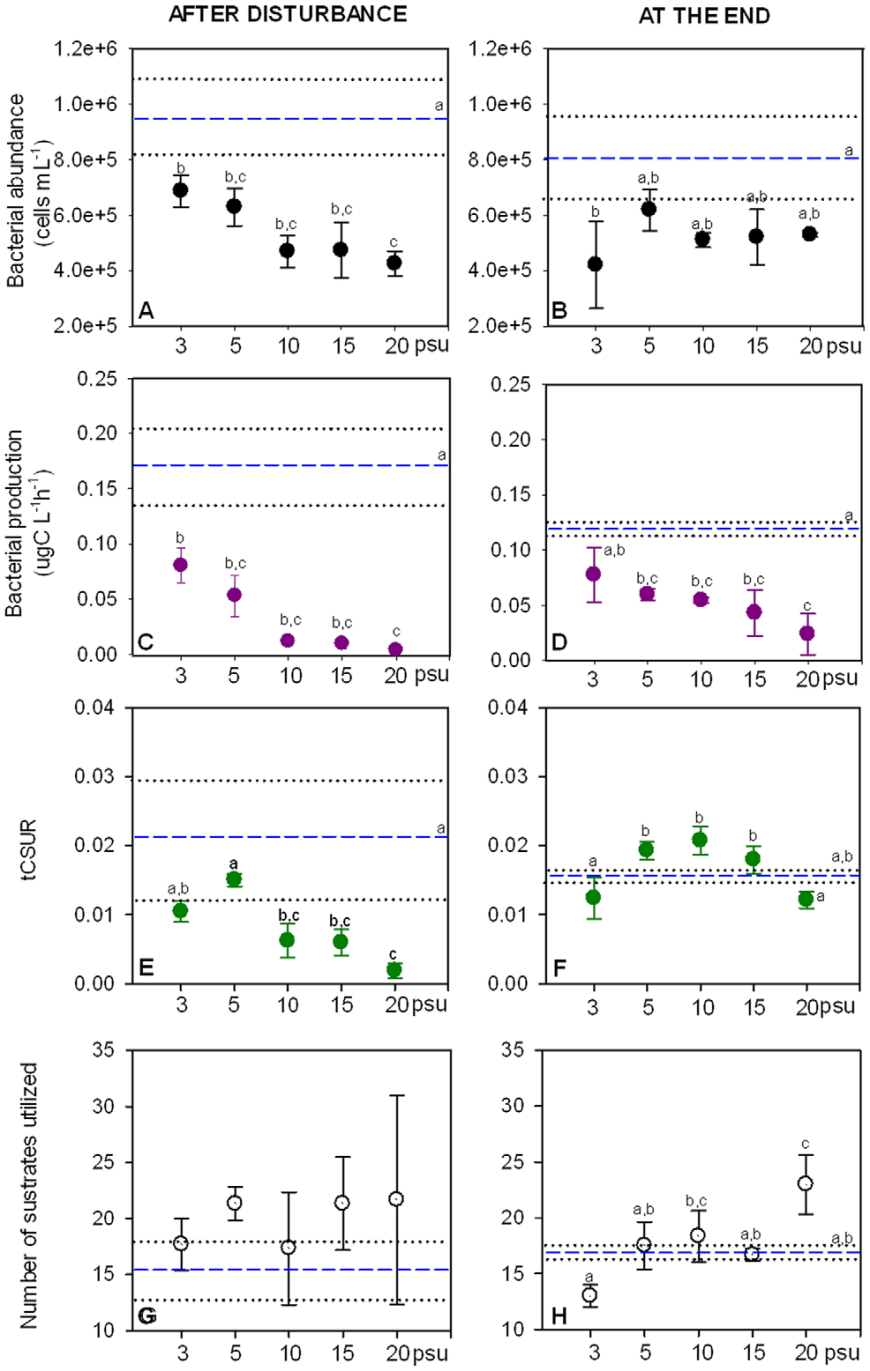
Functional changes in the disturbance intensity experiment (Experiment 1). Values presented correspond to mean values ± standard deviation of bacterial abundance (A, B), bacterial production (C, D), total carbon substrate utilization rate (tCSUR; E, F) and number of substrates with a positive response (G, H) for the different treatments. Mean values of controls are represented as dashed lines (- -), while standard deviation from control are represented as dotted lines (···). Tukey's homogenous groups are represented by a, b and c.

## Results

### Bacterial community composition

#### Number of TRFs

The number of TRFs decreased significantly with increasing disturbance intensity both after the disturbance and at the end of the experiment (F: 3.630, p<0.05, and F: 3.915, p<0.05, respectively), with lower number of TRFs at the 10 to 20 psu treatments. However, increases of disturbance frequency did not lead to significant changes of the number of TRFs at any sampling time (F:0.783, p>0.05 and F:1.074, p>0.05), after disturbance and at the end, respectively (Table S1).

#### Changes in Community composition

Active bacterial community composition did not show significant changes with increasing disturbance intensity (Experiment 1) directly after the disturbance (ANOSIM: R: 0.2, p>0.05), even though the high salinity treatments were clearly separated from the control and the low salinity treatments (Fig. 1A). At the end of the experiment, however, BCC differed significantly between treatments; high and intermediate intensities were separated from the control and the lowest intensity along the first dimension (ANOSIM: R: 0.31, p<0.05; Fig. 1B). For disturbance frequency (Experiment 2), differences in BCC were significant, both, directly after the disturbance (ANOSIM R: 0.53, p<0.001; Fig. 2A), as well as the end of the experiment (ANOSIM R: 0.55, p<0.001; Fig. 2B). Also here, the high frequency treatments differed from the control and the treatment with lowest frequency (1x) along both dimensions of the NMDS (Fig. 2A and 2B).

**Figure 4:**
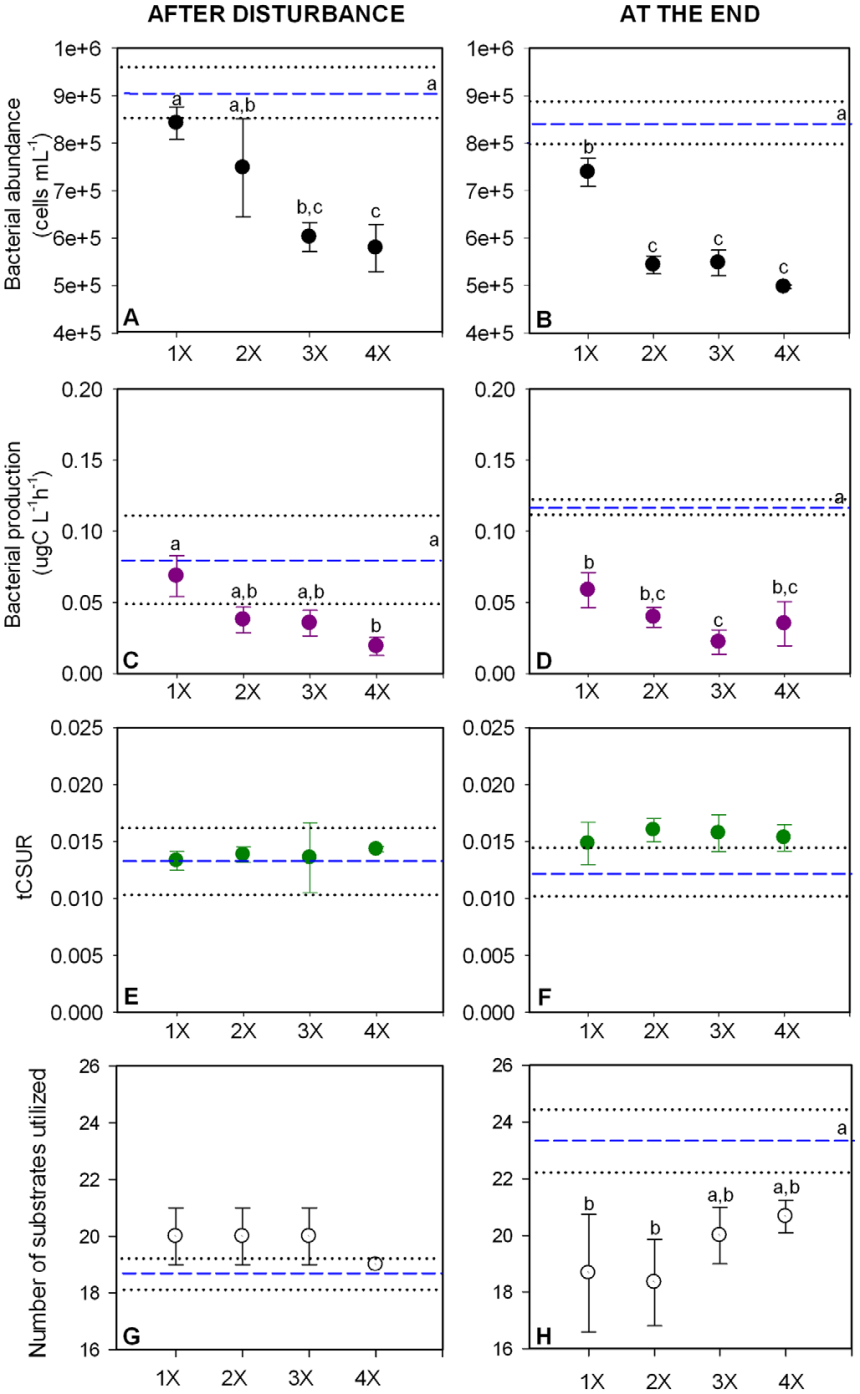
Functional changes in the disturbance frequency experiment (Experiment 2). Values presented correspond to mean values ± standard deviation of bacterial abundance (A, B), bacterial production (C, D), total carbon substrate utilization rate (tCSUR; E, F) and number of substrates with a positive response (G, H) for the different treatments. Mean values of controls are represented as dashed lines (- -), while standard deviation from control are represented as dotted lines (···). Tukey's homogenous groups are represented by a, b and c.

### Functions

#### Bacterial abundance and production

In the disturbance intensity experiment, bacterial abundance decreased significantly compared to the control with increasing salinity directly after the disturbance (F: 15.324, p<0.001; Fig. 3A). At the end, the changes were not as gradual but some of the treatments still differed from the control (F: 4.825, p<0.05; Fig. 3B). However, most of the treatments had abundances closer to the control value at the end of the experiment (36±9% of average decline in respect to the control) than they had directly after the disturbance (44±12% decline), suggesting a tendency to recover. Very similar patterns were observed for bacterial production, both, after the disturbance and at the end of the experiment (F: 17.278, p<0.001; Fig. 3C) and (F: 12.017, p<0.001; Fig. 3D), respectively, with a pronounced gradual decrease with increasing disturbance intensity. Bacterial production declined on average 81±20% compared to the control treatment directly after the disturbance and partly recovered towards the end of the experiment where the average decline was only 56±17%.

In the case of disturbance frequency (Experiment 2), bacterial abundance was gradually reduced by increasing the number of disturbance events and was significantly lower at the highest frequencies (3x and 4x; F: 17.251, p<0.001), whereas a single perturbation event did not cause significant changes (Fig. 4A). The differences between the high frequency treatments and the low frequency and the control treatment became larger at the end of the experiment (F: 79.890, p<0.001; Fig. 4B). Bacterial abundance declined, on average and compared to the control, 24±14% after the disturbance and further decreased to 31±13 % at the end of the experiment (Fig. 4A and 4B). A similar pattern was observed for bacterial production, although significant changes occurred only at the highest frequency of disturbance (4x; F: 7.214, p<0.01; Fig. 4C). Production rates were much lower compared to the control at the end of the experiment (F: 36.585, p<0.001) than they were after the disturbance and they differed significantly from the control for all the frequencies (Fig. 4D). Bacterial production declined on average 48±26% from the control after the disturbance but was reduced to 66±13% at the end of the experiment (Fig. 4C and 4D)

#### Carbon substrates utilization rates and number of positive substrates

Increases in disturbance intensity (Experiment 1) significantly decreased tCSURs after the disturbance (F: 14.920, p<0.001), with the exception of the lowest salinity treatments (3 and 5 psu) that showed a rate similar to the control (Fig. 3E). At the end of the experiment, no significant differences compared to the control were observed in any of the treatments. However, intermediate salinities (5–15 psu) showed significantly higher tCSURs than the treatments with the lowest and highest disturbances intensities (3 psu and 20 psu) (F: 9.281, p<0.01; Fig. 3F). tCSURs declined on average 62±24% compared to the control after the disturbance but resulted in an average increase (in respect to the control) of 13±23 % at the end of the experiment. After the disturbance, the number of positive substrates did not change significantly with increasing disturbance intensity (F: 0.904, p>0.05; Fig. 3G). At the end of the experiment there was a significantly higher number of positive substrates in the 20 psu treatment compared to the control (F: 10.677, p<0.01; Fig. 3H).

In experiment 2, tCSURs, in contrast to bacterial abundance and production, was not significantly affected by disturbance frequency (F_after disturbance_: 0.152, p>0.05 and F_end_: 0.152, p >0.05; Fig. 4E and 4F). The number of positive substrates did not show any significant change among treatments after the disturbance (F: 2.406, p>0.05; Fig. 4G). Significant differences were observed at the end of the experiment; the lowest frequency had significantly less positive substrates than the control and the higher frequency treatments (F: 6.393, p<0.01; Fig. 4H).

#### Utilization of carbon substrate guilds

**Figure 5:**
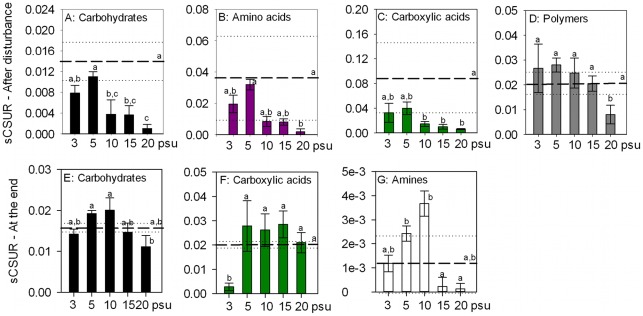
Changes in the average slope of significant substrate guilds in the intensity experiment (Experiment 1). Values plotted are the mean values ± standard deviation, after the disturbance (A–D) and at the end of the experiment (E–G). Mean values of controls are represented as dashed lines (- -) and standard deviations as dotted lines (···). Tukey's homogenous groups are represented by a, b and c.

In experiment 1, the substrates that were significantly influenced by differences in disturbance intensity directly after the disturbance belonged to carbohydrates, amino acids, carboxylic acids and polymers. The patterns of the average slopes of the different guilds were similar, however, differences between the treatments were observed (F_carbohydrates_: 12.861, p<0.001; F_amino acids_: 6.530, p<0.01; F_carboxylic acids_: 7.182, p<0.01 and F_polymers_: 6.858, p<0.01). In general rates decreased with increasing disturbance intensity with the exception of the 5 psu treatment that showed relatively higher rates than the other treatments (Fig. 5A–D). The number of significant guilds was reduced at the end of the experiment and comprised carbohydrates, carboxylic acids and amines. The average slope of carbohydrates, carboxylic acids and amines differed significantly between treatments (F_carbohydrates_: 6.242, p<0.01; F_carboxylic acids_: 17.581, p<0.001 and F_amines_: 7.151, p<0.01), although in this situation most of the treatments showed higher or similar rates compared to the controls. However, the patterns differed between the guilds (Fig. 5E–G).

**Figure 6:**
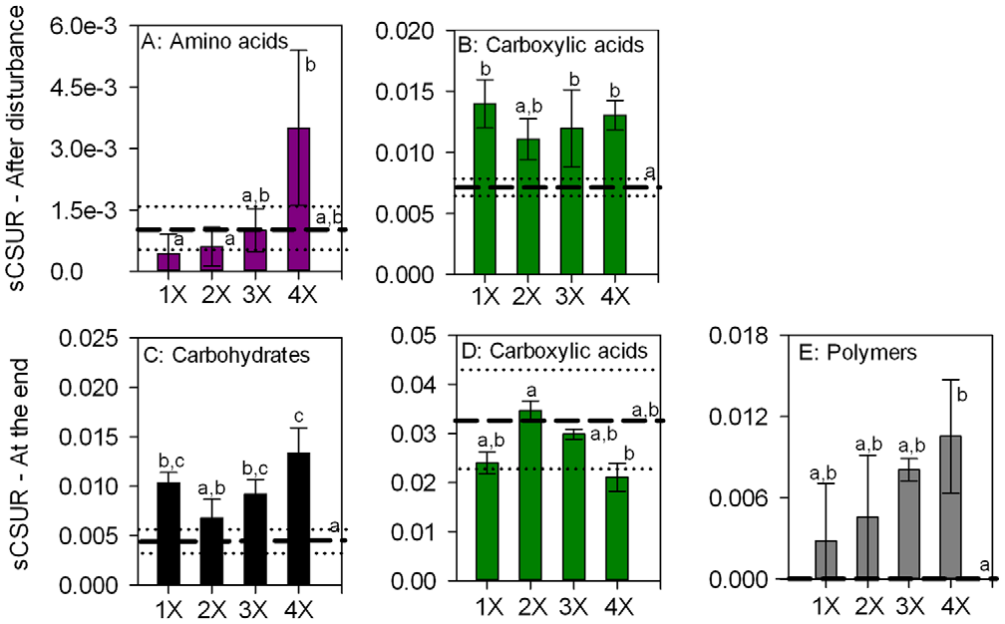
Changes in the average slope of significant substrate guilds in the intensity experiment (Experiment 2). Values plotted are the mean values ± standard deviation, after the disturbance (A–D) and at the end of the experiment (E–G). Mean values of controls are represented as dashed lines (- -) and standard deviations as dotted lines (···). Tukey's homogenous groups are represented by a, b and c.

In experiment 2 amino acids and carboxylic acids were the guilds that contained significant substrates directly after the disturbance. The average slope of amino acids differed between treatments (F: 4.899, p<0.05) being highest at the highest frequency (Fig. 6A). The slope of carboxylic acids was significantly increased by applying a disturbance, since all the levels of disturbance frequency showed higher slopes than the control (F: 6.861, p≤0.01; Fig. 6B). At the end the average slope of carbohydrates, carboxylic acids and the polymers differed significantly between treatments (F_carbohydrates_: 11.308, p<0.01; F_carboxylic acids_: 4.134, p<0.05 and F_polymers_: 5.587, p<0.05), showing an increase with increasing frequency, with exception of the carboxylic acids that peaked in the 2x treatment and had in general rather low rates compared to the control (Fig. 6C–E).

### BCC and function parameters correlations: Mantel test

In experiment 1 there were significant, albeit weak, correlations between BCC (Bray-Curtis distances) and the overall bacterial production, both directly after the disturbance and at the end of the experiment. No significant correlation was observed between BCC and tCSURs at any time point. However, significant correlations were observed between BCC and significant sCSURs (Table 1).

**Table 1:** Mantel test results.

			BCC
Exp. 1: Intensity	After disturbance	Bacterial production	**0.342, p<0.0001**
		tCSUR	0.073, p: 0.3881
		Significant sCSUR	**0.351, p: 0.0003**
	At the end	Bacterial production	**0.361, p<0.0001**
		tCSUR	0.000, p: 0.9909
		Significant sCSUR	**0.255, p: 0.0041**
Exp. 2: Frequency	After disturbance	Bacterial production	**0.613, p<0.0001**
		tCSUR	**0.339, p: 0.0021**
		Significant sCSUR	−0.024, p: 0.8245
	At the end	Bacterial production	**0.293, p: 0.0039**
		tCSUR	0.045, p: 0.6307
		Significant sCSUR	0.024, p: 0.7873

Presented are the r^M^ values and the significance levels for correlations between bacterial community composition (BCC), bacterial production, total substrate utilization rates (tCSUR) and the rates of utilization of significant substrate guilds (significant sCSUR). Bold text indicates significant correlations (p≤0.05).

For disturbance frequency (Experiment 2), BCC was significantly correlated with both, overall bacterial production and tCSUR after the disturbance, although the correlation was weaker in the latter case. At the end of the experiment, BCC was only significantly correlated to the overall bacterial production. Significant substrates were not correlated with BCC at any of the time points (Table 1).

## Discussion

It has been shown that bacterial communities are often affected by disturbances [17], [37]–[39], but it is likely that these response patterns are influenced by the strength of a disturbance, such as its intensity and frequency. Here we show that stronger changes in community composition result from an increase in disturbance strength, both when considering the intensity and frequency of a disturbance and that a similar pattern was observed only for some of the studied functions.

Our study shows that disturbance strength has the potential to change the functional performance and composition of bacterial communities and confirms previous findings that bacterial communities are generally not resistant to disturbances [40]. Moreover, the response pattern was primarily gradual in character, i.e. the stronger the disturbance the stronger was the change in BCC suggesting a positive relationship. A similar pattern has also been observed in other studies [41]–[44] when studying diversity-disturbance relationships in larger organisms. However, the response patterns can vary widely [1] since also no [45] or bell-shaped [46], [47] relationships have been previously found. The fact that no clear and consistent predictive pattern can be observed in disturbance-diversity relationships has been attributed to the fact that various traits such as intrinsic growth rates, competition, predation and environment productivity influence the relationship [48].

In both experiments we found that different functions showed differences in their response patterns to increasing disturbance strengths. Abundance and production decreased with increasing disturbance strength, probably due to direct physiological effects of salinity on bacteria which reduces their growth rates [19], [49]. This is also in congruence with previous studies reporting similar effects of disturbances on abundance, biomass and productivity of other organism groups [50]–[52]. In the intensity experiment, bacterial abundance and production had a tendency to recover at all levels by the end of the experiment, in particular in most of the low disturbance intensity treatments, but not in the frequency experiment. Even though it is not possible to compare the two experiments directly, these results nevertheless show clearly that effects of disturbances on communities are highly context dependent, which is also in agreement with the wide range of different response patterns observed in the literature mentioned above for macroorganisms.

The tCSURs showed a different pattern than bacterial abundance and production and were not at all affected by different disturbance frequencies. This might have been the result of a combined effect of the number of substrates utilized and the rates at which they were used. More specifically, at the end of the experiment, communities exposed to low frequencies tended to use relatively few substrates, but they probably used them at high rates resulting in tCSUR values similar to those found in communities exposed to high frequencies where, on the contrary, more substrates were used, but probably at lower rates. On the contrary, very variable response patterns were observed regarding the significant substrate guilds, ranging from gradual decreases, bell shape responses or even increases, therefore effects on the different guilds are difficult to predict. At the end of the experiment, tCSURs showed a bell-shaped response pattern to differences in the applied disturbance intensities, indicating that negative effects were strongest at low and high disturbance intensities and that intermediate levels of disturbance may enhance tCSURs. This is in congruence with predictions made by the intermediate disturbance hypothesis [53] that suggests that highest diversity is maintained at intermediate disturbance levels. We further observed, that at least for some substrates guilds, higher utilisation rates also occurred at intermediate disturbance intensities, providing further support that certain community functions peak at intermediate rates of disturbance intensity. The tCSUR results also show that there was a remarkably fast recovery of the overall substrate utilization rates, which in some cases even exceeded the control values at the end of the experiment, supporting the idea that disturbances might enhance community function under some circumstances [54], [55].

The fast recovery and/or overcompensation (Experiment 1), the general lack of sensitivity of tCSURs (Experiment 2) as well as the increase in the number of utilized substrates (both experiments, see above) with increasing disturbance strength might be related to the increase of disturbance tolerant, ‘weedy’, fast growing taxa that became dominant at high disturbance strengths [48], [56]. Alternatively, disturbance-induced changes in community composition may have modified the extend and character of competitive interactions among taxa in the community resulting in new selective pressures which ‘forced’ taxa to widen their substrate range or switch to other substrates, resulting in an overall increase in the number and rates with which they were utilized.

Even though most of the measured functions were affected by increases in disturbance strength, an important conclusion of this study is that the overall effects and rates of recovery varied between the different functions. Other examples of this phenomena have been reported [19], [54]. Furthermore, differences between patterns of different functions have been related to the fact that they might assess different levels of the community (from individual cells to community level) [15] or depend on how specific the function is [16], [57]. In our study important differences were observed between the patterns described by bacterial production and tCSURs. Bacterial production measurements are based on the incorporation of a single widely available substrate, whereas tCSUR incorporates several and also more specific substrates. Moreover, differences might also be of methodological nature, since bacterial production is a short-term measurement of the *in situ* activity of the communities, whereas tCSUR measurements involve a cultivation step and do therefore rather provide a long-term metabolic fingerprinting.

As a further result of the varying response patterns of different functions to increasing disturbance strength, correlations between BCC and functions showed a high degree of differences as well. In general, higher correlations were observed between BCC and bacterial production than between BCC and tCSUR or the number of utilized substrate guilds. This indicates that the use of a single, widely used substrate, such as leucine, better follows the patterns observed in BCC than composite functional measurements based on a variety of substrates, which instead lead to more complex responses and therefore a wider variety of patterns.

Interestingly correlations between community composition and functions were in most cases, when occurring, rather weak. The fact that we did not observe stronger relationships might be due to the following reasons: (1) plasticity [58], (2) functional redundancy [59] or (3) due to a time lag in the responses. Our results suggest that functional redundancy was important for some functions that either did not change at all after the disturbance (e.g. tCSURs in frequency treatment) or were similar to the unperturbed control at the end of the experiment (e.g. tCSUR in intensity experiment) even though there were persistent changes in composition. It has, however, been pointed out that the relationship between community composition and functioning is very dynamic and that absolute patterns between community composition and functions are often not correlated whereas their rates of changes along environmental gradients are [59]. This is similar to what we observed here since we also found weak correlations regarding the absolute patterns, but often, strong changes in both community composition and functions along the disturbance gradients.

There are also a few methodological issues that could explain the overall weak relationships between community composition and functions. It is well documented that T-RFLP is a method that detects only the most abundant taxa in a community [28] and therefore might miss rare taxa that are functionally important. Moreover, there are recent studies that show that functional traits or genes might be better suited than species composition to explain the assembly and functioning of bacterial communities [60], [61]. In the study of Burke et al. [61], for example, complex host-associated microbial communities had a consistent pool of functional genes whereas taxa-level community composition was highly variable. Thus, future studies should investigate whether we see stronger links between composition and functioning when looking at functional groups instead of taxa.

Moreover, asynchronies in response patterns were observed in the intensity experiment where clear functional changes occurred already after the disturbance period, whereas changes in the composition of the active community were not significant until the end of the experiment, which is in congruence with other studies that showed that there is often a higher sensitivity in functional measurements than in community composition [39]. A second explanation could be that the salinity disturbance had a strong filtering effect leading to the selection of species with a wide range of salt tolerance that then further increased in activity and abundance once the disturbance had ceased. In case of the frequency experiment, changes in function and composition occurred more synchronized in direct response to the disturbance. However, in this case disturbance periods were shorter and the communities were regularly exposed to freshwater conditions in between the disturbance(s), which might have induced more pronounced shifts in community composition already during the disturbance period. Previous experiments have reported that both functions [62]–[65], as well as community composition [10], [37] can change over relatively short time periods, i.e. within hours or days, but there are, as in our study, also observations of slower changes in BCC compared to bacterial activity after pesticide addition on sediments [39] or asynchronous changes at community level physiological profiles (CLPP) and genotype levels after different management treatments in agricultural soil [66]. One possible explanation for such asynchronies in our study could be that active BCC and bacterial production detect the *in situ* situation whereas tCSURs are obtained after an incubation step, which might explain why higher correlations were observed between BCC and production than between BCC and tCSURs.

Our study shows, moreover, that disturbance responses can be case dependent since the lag between the functional and compositional response was only observed in the intensity but not in the frequency experiment. It also highlights time as a generally very important aspect when studying disturbances and how bacterial communities respond to them. In particular, to properly address resilience in functional properties as well as community composition longer recovery times and more sampling points would be required during and particularly after the perturbation to be able to describe different states, e.g. a partly altered, maximal altered, and, if applicably, a partly recovered and totally recovered community.

To summarize, our study suggests that (1) bacterial community composition changes gradually along disturbance gradients, showing no resistance to any level of salinity intensity or frequency. The response pattern to changes in disturbance strength varied depending on the type of function and differed in response to changes in disturbance intensity or frequency, respectively. Further, (2) the potential recovery of BCC could not adequately be assessed, however, there were some indications that communities recovered functionally to greater extents from low compared to medium and high disturbance intensities, whereas no tendency of short term recovery was observed in response to manipulations of disturbance frequencies. Finally, (3) whether congruent response patterns in BCC and functions along the disturbance gradients were observed depended on the type of function and the type of disturbance, respectively. Our results therefore highlight that effects of disturbances on the resistance and resilience of bacterial communities are complex and highly context dependent, which is something future studies need to take into account.

## Supporting Information

Table S1 **Number of T-RFs.** Presented is the average number of the TRFs ± standard deviation, for each treatment, time point and experiment.
(DOCX)
