# Supplementary material for: Effects of Disturbance Intensity and Frequency on Bacterial Community Composition and Function
Source: PLoS One. 2012 May 14;7(5):e36959. doi: 10.1371/journal.pone.0036959 (PMC3351442; doi:10.1371/journal.pone.0036959)
Supplement: Table S1 — Number of T-RFs. Presented is the average number of the TRFs ± standard deviation, for each treatment, time point and experiment. (DOCX) [file pone.0036959.s001.docx]

|  | After disturbance | | | At the end | |  |  |
| --- | --- | --- | --- | --- | --- | --- | --- |
| Disturbance intensity | Num. T-RFs |  |  | Num. T-RFs | | |  |
| Control | 18,0 | ± | 0,0 | 19,3 | ± | | 2,1 |
| 3 psu | 17,7 | ± | 1,5 | 18,0 | ± | | 1,0 |
| 5 psu | 16,0 | ± | 4,2 | 19,0 | ± | | 1,4 |
| 10 psu | 14,3 | ± | 1,5 | 15,7 | ± | | 2,3 |
| 15 psu | 12,7 | ± | 1,2 | 14,3 | ± | | 0,6 |
| 20 psu | 13,3 | ± | 1,5 | 16,0 | ± | | 2,0 |
| Disturbance Frequency | Num. T-RFs |  |  | Num. T-RFs | | |  |
| Control | 15,3 | ± | 2,8 | 16,7 | ± | | 0,6 |
| 1X | 16,0 | ± | 0,0 | 16,3 | ± | | 0,6 |
| 2X | 14,0 | ± | 2,0 | 15,7 | ± | | 3,2 |
| 3X | 14,0 | ± | 2,0 | 14,3 | ± | | 0,6 |
| 4X | 15,0 | ± | 0,0 | 16,0 | ± | | 0,0 |
